# Gheel.—Letter from Dr. Willers Jessen

**Published:** 1860-10-01

**Authors:** Willers Jessen

**Affiliations:** Hornhiem, near Kiel


### GHEEL.—LETTER FROM DR. WILLERS JESSEN.

(*To the Editor of the Journal of Psychological Medicine.*)

Sir,—In the last number of your Journal, of which I have been a reader for several years, I find an article directed partly against myself, partly, with regard to my quotations, against my English co-thinkers. I pass in silence the offensive personalities addressed to myself as being without justification, and consequently proving nothing but bad taste. Neither do I think proper to oppose assertions consisting only in vague phrases, unsupported by any arguments. If Dr. Parigot calls the medical officers of asylums dreamers, psychologists, and mad doctors, and praises St. Dymphna, if he represents the asylums as prisons, and speaks of liberty and non-restraint in Gheel, where the sick people are put in "fetters, chains, and irons," he will no doubt produce the most disadvantageous impression upon your countrymen, who have established the best asylums of the world, and who are the last to be deceived by sophisms.

I therefore shall only blame the great want of exactness with which he repeats my expressions. My best argument is, he says, that "Gheel ought to be a practical criticism upon asylums;" but I never have made use of such a phrase. I have tried to prove by facts that no organization answering to the purpose could be given to lunatic colonies; I have insisted that no such colonies ought to be founded, before a possibility could be shown to avoid the improprieties Dr. Parigot himself has often indicated. If he had afforded this proof, I should gratefully have accepted his reply; as he has not even tried to do so, I must believe that he is incapable of it.

In the same incorrect way he says: "Following an article by Dr. W. Jessen, we find that Dr. Bucknill compares Gheel to the small English asylums, which he calls, with reason, squalid asylums." As to the last expression, Dr. Bucknill has only used it of Gheel, and has asserted on the contrary that the same reasons would be *justly* applied to Gheel, that have "so *unjustly* been urged" against private lunatic asylums. I have translated Dr. Bucknill word for word, and have given no occasion to Dr. Parigot's spiteful remarks on the English private establishments.

Finally, he writes: "In a paper which is quoted by the *Allgemeine Zeitschrift*, Dr. Stevens asserts that my honourable successor, Dr. Bulckens, told him that he did not possess any means of controlling the exorcisms practised in the chapel of St. Dymphna; that if it was in his power to put a stop to them, he should not think it prudent to do so, because what constitutes the colony is not medical science, but faith in St. Dymphna; and that if the saint disappeared, or was neglected, *Gheel would have no more cause to exist.*" "Unfortunately, however," he adds soon after, "Dr. Bulckens affirms, and we have no difficulty in believing him, that he said nothing of the kind. Dr. Stevens, doubtless from want of familiarity with the French language, has evidently misunderstood what was said to him, and even what he saw." To my translation I had added the following passage of the original text in English: "As rev erence for Dymphna, the presiding saint, and no faith in medicine, ruled the colony, and he thought that, Dymphna once ignored or slighted, *but little of Gheel, as a means of harbouring the insane, would remain.*" But now, Dr. Parigot has addressed to Dr. Droste in Osnabruck a letter, part of which the latter has put in print. In a journal edited by himself, *Medicinische Aehrenlese*, (January, 1860, No. 1,) there is to be read as follows: " J'ai lu l'article indignant (?) de M. Willers Jessen dans le cahier d'Octobre de *l'Allgemeine Zeitschrift für Psychiatrie*, &c., de 1859. M. Bulckens a dit à MM. Bucknill et Stevens: Si l'on n'adorait plus Sainte Dymphna, Gheel n'aurait plus de raison d'être." After this, Dr. Parigot, in his quotation, has thus changed the original text of Dr. Stevens, that at the conclusion he agrees literally with the expression, which, according to his letter, Dr. Bulckens has actually made. It is incomprehensible how he can affirm, notwithstanding, that the latter has said "nothing of the kind;" but I will not decide against Dr. Parigot's trustworthiness, till he has given himself an explanation of this most striking contradiction.

I am, Sir, yours obediently,

Hornhiem, near Kiel, Dr. WILLERS JESSEN.

July the 23rd, 1860.

